# *Bta-miR-484* Targets *SFRP1* and Affects Preadipocytes Proliferation, Differentiation, and Apoptosis

**DOI:** 10.3390/ijms241612710

**Published:** 2023-08-11

**Authors:** Mengli Yang, Xiaoqian Gao, Chunli Hu, Shuzhe Wang, Hui Sheng, Yun Ma

**Affiliations:** Key Laboratory of Ruminant Molecular and Cellular Breeding of Ningxia Hui Autonomous Region, College of Animal Science and Technology, Ningxia University, Yinchuan 750021, China

**Keywords:** *bta-miR-484*, *SFRP1*, bovine preadipocytes, differentiation, proliferation

## Abstract

MicroRNAs (miRNAs) are essential regulators of numerous biological processes in animals, including adipogenesis. Despite the abundance of miRNAs associated with adipogenesis, their exact mechanisms of action remain largely unknown. Our study highlights the role of *bta-miR-484* as a major regulator of adipocyte proliferation, apoptosis, and differentiation. Here, we demonstrated that the expression of *bta-miR-484* initially increased during adipogenesis before decreasing. Overexpression of *bta-miR-484* in adipocytes ultimately inhibited cell proliferation and differentiation, reduced the number of EdU fluorescence-stained cells, increased the number of G1 phase cells, reduced the number of G2 and S phase cells, and downregulated the expression of proliferation markers (*CDK2* and *PCNA*) and differentiation markers (*CEBPA*, *FABP4*, and *LPL*). Additionally, overexpression of *bta-miR-484* promoted the expression of apoptosis-related genes (*Caspase 3*, *Caspase 9*, and *BAX*), and increased the number of apoptotic cells observed via flow cytometry. In contrast, *bta-miR-484* inhibition in adipocytes yielded opposite effects to those observed during *bta-miR-484* overexpression. Moreover, luciferase reporter assays confirmed *SFRP1* as a target gene of *bta-miR-484*, and revealed that *bta-miR-484* downregulates *SFRP1* mRNA expression. These findings offer compelling evidence that *bta-miR-484* targets *SFRP1*, inhibits proliferation and differentiation, and promotes apoptosis. Therefore, these results offer novel insights into the *bta-miR-484* regulation of adipocyte growth and development.

## 1. Introduction

Lipogenesis was the process by which preadipocytes differentiate into mature adipocytes, characterized by an increased number of adipocytes and increased lipid storage in adipocytes, which controls the renewal of adipocytes throughout the life cycle. Adipogenesis was a complex network regulatory process mediated by a series of regulatory factors. Current studies have demonstrated that microRNA (miRNA) was involved in lipid metabolism and adipogenesis. Specifically, *bta-miR-193b* [1] and *miRNA-204-5p* [2] both regulate adipocyte differentiation, proliferation, and apoptosis. *miR-146a* [3], *miR-222-3p* [4], and *microRNA-27b* [5] promote preadipocyte differentiation by targeting recombinant natriuretic peptide receptor 3 (*NPR3*), DNA-damage-inducible transcript 4 (*DDIT4*), and acyl-CoA thioesteras 2 (*ACOT2*). Additionally, *miR-26* [6] and *miR-107* [7] inhibit adipocyte differentiation and adipogenesis by targeting F-box and leucine-rich repeat protein 19 (*FBXL19*) and cyclin-dependent kinase 6 (*CDK6*). Alternatively, *bta-miR-150* [8] and *bta-miR-2400* [9] have been determined to promote bovine adipocyte proliferation and inhibit differentiation. *Bta-miR-149-5p* [10] and *bta-miR-376a* [11] target *CREB* regulated transcription coactivator (*CRTC*) and KLF transcription factor 15 (*KLF15*), which inhibits the proliferation and differentiation of bovine adipocytes. Despite this progress, there remain several miRNAs that may participate in adipocyte differentiation and fat deposition, warranting further exploration into the mechanisms of these miRNAs in adipogenesis.

*miR-484* has been implicated in the regulation of a wide range of biological processes, including dengue virus replication [12], acute coronary syndrome [13], multiple sclerosis [14], liver cirrhosis and fibrosis [15], pulmonary fibrosis [16], prostate cancer [17], ovarian cancer [18], metastatic renal cell carcinoma [19], gastric cancer [20], and mental illnesses [21,22]. Furthermore, *bta-miR-484* has been predicted to possibly down-regulate the human heart fatty acid binding protein 3 (*FABP3*) gene and thus intramuscular fat deposition [23]. In the present study, we evaluate the potential relationship between *bta-miR-484* and adipocyte differentiation in the laboratory. *Bta-miR-484* expression was detected at different differentiation stages in bovine preadipocytes; this expression exhibited an initial upward trend before a subsequent downward trend, which was hypothesized to be associated with the adipocyte differentiation process. However, the function and mechanism of action of *bta-miR-484* in bovine adipocyte proliferation, apoptosis, and differentiation remain largely unexplored. Therefore, in this study, *bta-miR-484* was overexpressed or knocked down in bovine preadipocytes to explore the regulatory mechanisms of *bta-miR-484* in these biological processes. Further, a dual-luciferase reporter assay verified whether *bta-miR-484* has a targeting relationship with the secreted frizzled-related protein 1 (*SFRP1*) gene, providing a theoretical basis for further perfecting the molecular network of miRNA regulation of adipogenesis.

## 2. Results

### 2.1. Temporal Expression Profile of bta-miR-484 during Adipose Differentiation

**Figure 1:**
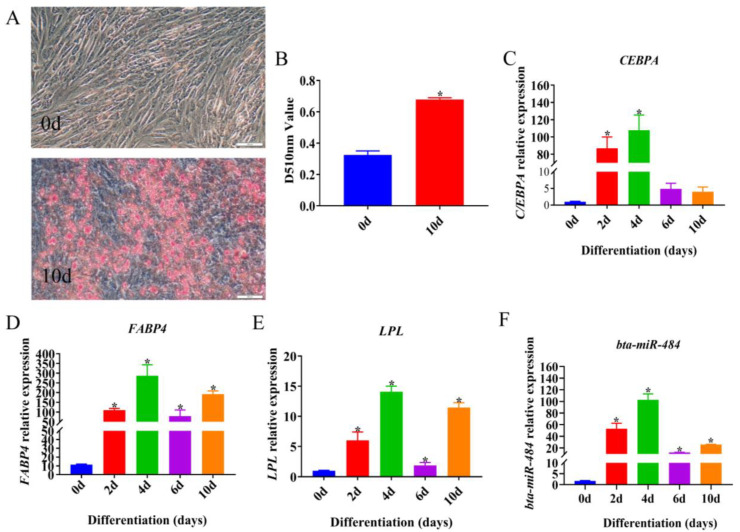
Induced preadipocytes differentiation. (**A**) Oil Red O staining at 0 d and 10 d after induced preadipocytes differentiation. Scale bar: 200 μm. (**B**) Oil Red O absorption value detected at 510 nm. (**C**–**F**) Expression of *CEBPA*, *FABP4*, *LPL,* and *bta-miR-484* during induction of preadipocyte differentiation. Data are presented as mean ± SD. n = 3. * *p* < 0.05.

In order to verify whether the isolated primary adipocytes could be successfully induced to differentiate, the expression levels of adipocyte differentiation marker genes CCAAT/enhancer binding protein alpha (*CEBPA*), Fatty acid-binding protein (*FABP4*), and lipoproteinlipase (*LPL*) were detected at different times of induction differentiation, and the cells were stained with Oil Red O 10 d after induction differentiation. The results demonstrated that the differentiation induced promoted the accumulation of lipid droplets within adipocytes, increased the absorption value of the Oil Red O staining solution eluted by isopropanol (Figure 1A,B), and promoted the expression of *CEBPA*, *FABP4*, and *LPL*, which were lipid markers (Figure 1C–E). These results revealed successful differentiation of the preadipocytes used in this study, affirming their suitability for subsequent experiments. The expression of *bta-miR-484* was then detected at different time points during the induction of preadipocyte differentiation. With an increase in induction time, the expression of *bta-miR-484* initially increased before decreasing, reaching its highest value on day 4 (Figure 1F).

### 2.2. Bta-miR-484 Inhibits Adipocyte Proliferation

**Figure 2:**
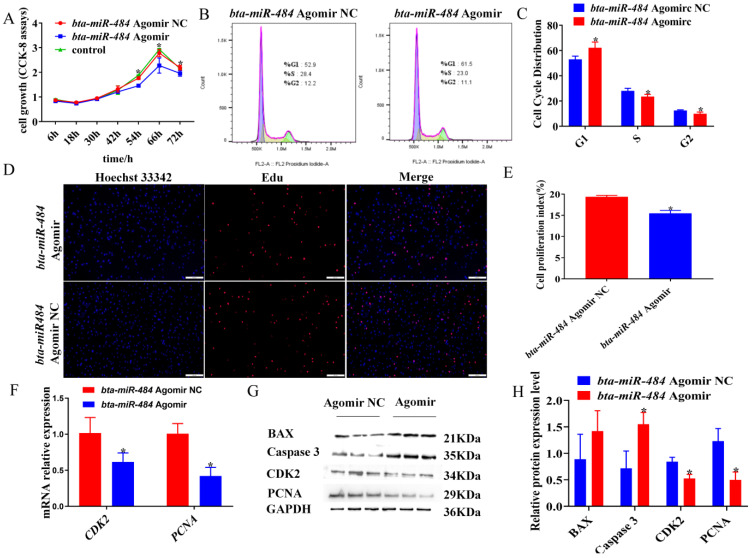
*Bta-miR-484* agomir inhibited the proliferation of preadipocytes. (**A**) CCK-8 assay to determine adipocyte viability. (**B**,**C**) Cell cycle phase analysis of adipocytes by flow cytometry. (**D**,**E**) Adipocyte proliferation was examined by EdU immunofluorescent staining. Red represents EdU staining; blue represents cell nuclei stained with Hoechst 33342. Scale bar: 500 μm. (**F**) Relative mRNA expression of *CDK2* and *PCNA* genes in preadipocytes 48 h after transfection with a *bta-miR-484* agomir or *bta-miR-484* agomir NC. (**G**,**H**) Protein expression levels of CDK2, PCNA, Bcl-2-associated X protein (BAX), and cysteine-dependent aspartate-specific proteases 3 (Caspase 3) were detected in *bta-miR-484* over-expressing adipocytes using WB. GAPDH was used as an internal reference. Data are presented as mean ± SD. n = 3. * *p* < 0.05.

**Figure 3:**
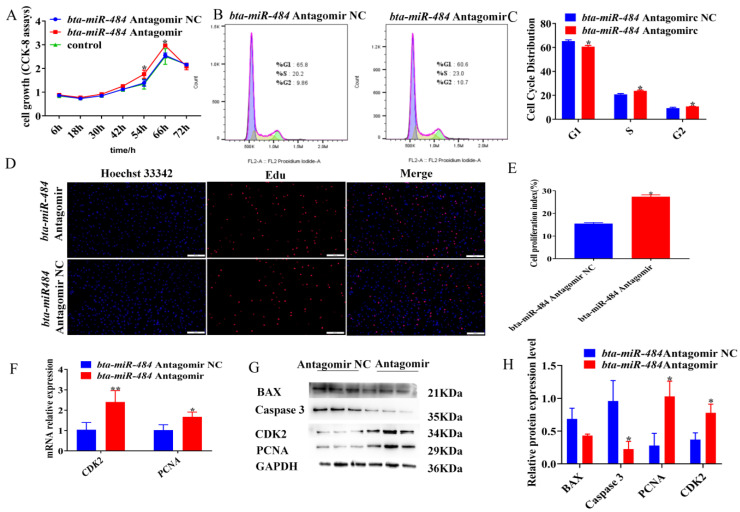
Inhibition of *bta-miR-484* promotes adipocyte proliferation. (**A**) CCK-8 assay to determine adipocyte viability. (**B**,**C**) Cell cycle phase analysis of adipocytes using flow cytometry. (**D**,**E**) Adipocyte proliferation was examined by EdU immunofluorescent staining. Red represents EdU staining; blue represents cell nuclei stained with Hoechst 33342. Scale bar: 500 μm. (**F**) Relative mRNA expression of *CDK2* and *PCNA* in preadipocytes 48 h after transfection with a *bta-miR-484* antagomir or *bta-miR-484* antagomir NC. (**G**,**H**) Protein expression levels of CDK2, PCNA, BAX, and Caspase3 were detected in *bta-miR-484* down-regulated adipocytes using WB. GAPDH was used as an internal reference. Data are presented as mean ± SD. n = 3. * *p* < 0.05. ** *p* < 0.01.

To investigate the role of *bta-miR-484* in adipocyte proliferation, preadipocytes were transfected with a *bta-miR-484* agomir, *bta-miR-484* agomir negative control (agomir NC), *bta-miR-484* antagomir, or *bta-miR-484* antagomir negative control (antagomir NC). Subsequently, the cell counting kit-8 (CCK-8) assay, 5-ethynyl-2’-deoxyuridine (EdU) assay, flow cytometry, real-time quantitative PCR detecting system (qPCR), and western blotting (WB) were used to assess the effect of *bta-miR-484* on the proliferation of adipocytes. Overall, transfection with the *bta-miR-484* agomir reduced the proliferative activity of adipocytes (Figure 2A), increased the number of cells in the G1 phase cells, reduced the number of S and G2 phase cells (Figure 2B,C), decreased the number of cells in a proliferative state (Figure 2D,E), and inhibited the gene and protein expression of cyclin-dependent kinase 2 (*CDK2*) and proliferating cell nuclear antigen (*PCNA*) (Figure 2F–H). In contrast, the *bta-miR-484* antagomir enhanced the proliferative activity of adipocytes (Figure 3A), decreased the number of G1 phase cells, increased the number of S and G2 phase cells (Figure 3B,C), increased the number of proliferative cells (Figure 3D,E), and promoted the genes and protein expression of CDK2 and PCNA (Figure 3F–H). Collectively, these findings indicated that *bta-miR-484* inhibited adipocyte proliferation.

### 2.3. Bta-miR-484 Promotes Adipocyte Apoptosis

**Figure 4:**
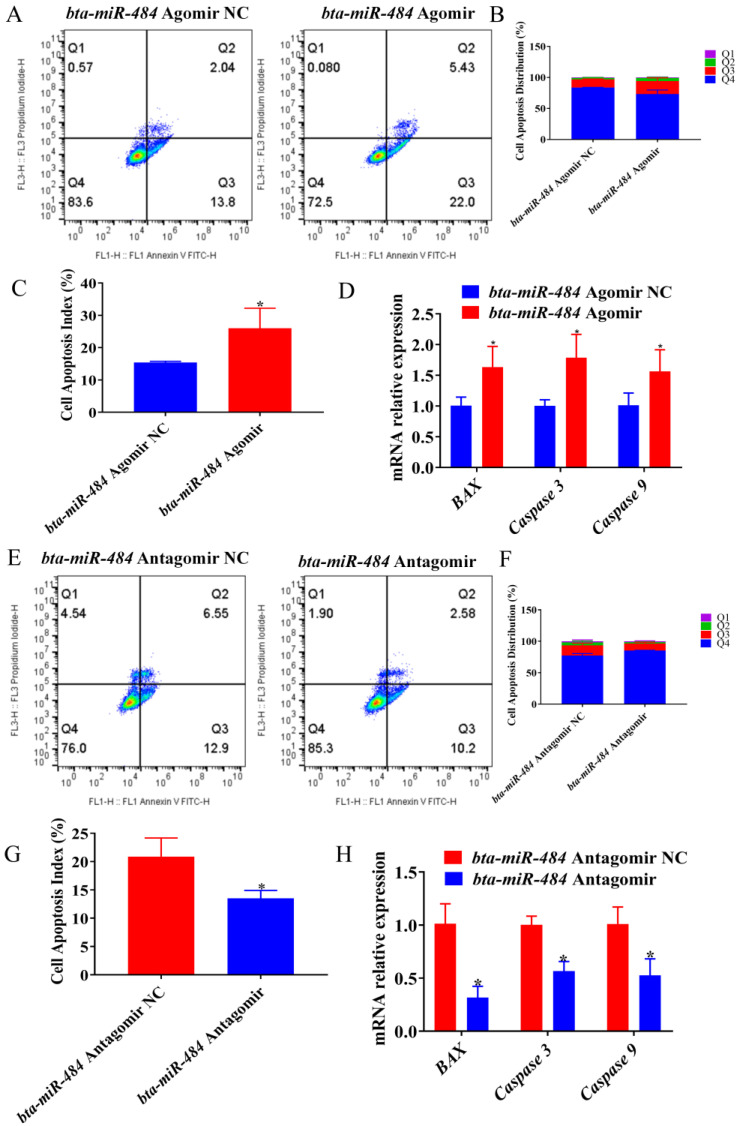
The effect of *bta-miR-484* overexpression or silencing on adipocyte apoptosis. (**A**–**C**,**E**–**G**) The apoptotic phase of adipocytes was analyzed using flow cytometry. (**D**,**H**) Relative mRNA expression of *BAX*, *Caspase 3*, and *Caspase 9* genes in *bta-miR-484* overexpressing or *bta-miR-484* inhibited adipocytes. Data are presented as mean ± SD. n = 3. * *p* < 0.05.

We then examined the effect of *bta-miR-484* on adipocyte apoptosis. First, we performed an annexin V-FITC/PI staining assay. Overall, overexpression of *bta-miR-484* was found to increase the apoptosis index of adipocytes (Figure 4A–C). Subsequently, we evaluated the mRNA expression of genes associated with cell survival by qPCR. The corresponding results demonstrated that overexpression of *bta-miR-484* increased *BAX*, *Caspase 3*, and cysteine-dependent aspartate-specific proteases 9 (*Caspase 9*) expression (Figure 4D). Finally, WB analysis showed enhanced expressions of BAX and Caspase 3 proteins in *bta-miR-484*-overexpressing adipocytes (Figure 2G,H). In contrast, silencing *bta-miR-484* reduced the apoptotic index of adipocytes, inhibited the expressions of the apoptosis-related marker genes *BAX*, *Caspase 3*, and *Caspase 9*, and decreased the expressions of BAX and Caspase 3 proteins (Figure 3G,H and Figure 4E–H). These results demonstrated that *bta-miR-484* promotes adipocyte apoptosis.

### 2.4. Bta-miR-484 Inhibits Adipocyte Differentiation

**Figure 5:**
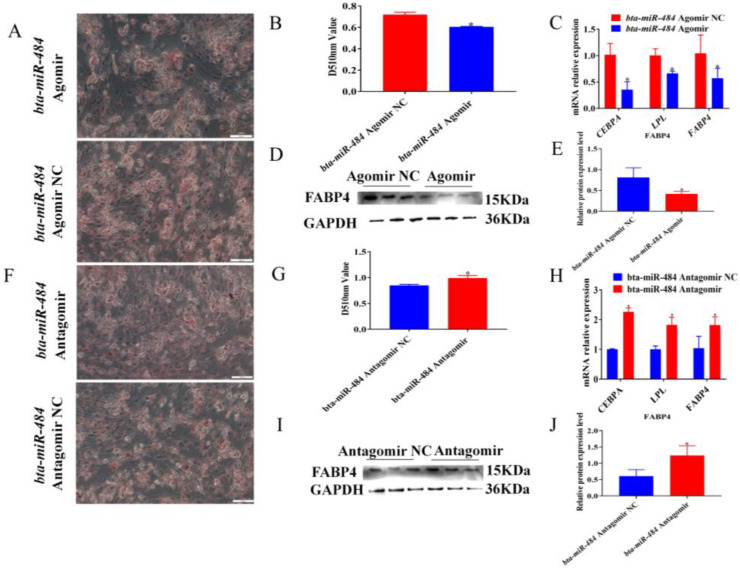
*Bta-miR-484* inhibits the differentiation of preadipocytes. (**A**,**F**) Graphs show brightfield microscopy of differentiated adipocytes stained by Oil Red O for *bta-miR-484* overexpression or inhibition adipocytes. Scale bars: 500 μm. (**B**,**G**) The Oil Red O dye was eluted with 100% isopropanol and the absorbance was detected at 510 nm. (**C**,**H**) Expression of *CEBPA*, *LPL*, and *FABP4* genes was measured 6 d after induction of differentiation in *bta-miR-484*- overexpressing or inhibiting adipocytes. (**D**,**E**,**I**,**J**) Protein levels of FABP4 were detected by the WB of adipocytes with *bta-miR-484* overexpression or inhibition. GAPDH was used as an internal reference. Data are presented as mean ± SD. n = 3. * *p* < 0.05.

To explore the role of *bta-miR-484* in adipocyte differentiation, *bta-miR-484* agomir, *bta-miR-484* agomir NC, *bta-miR-484* antagomir, or *bta-miR-484* antagomir NC were transfected into preadipocytes; then, 6 d after differentiation induction, the effects of *bta-miR-484* on lipid droplet accumulation and the expression of adipogenesis-associated genes and proteins were assessed using Oil Red O staining, qPCR, and WB. The Oil Red O staining results indicated that the *bta-miR-484* agomir inhibited the accumulation of lipid droplets and reduced the absorbance value of the Oil Red O dye eluted by isopropanol at 510 nm (Figure 5A,B). Additionally, the qPCR results demonstrated that the *bta-miR-484* agomir reduced the expression of *CEBPA*, *LPL*, and *FABP4* (Figure 5C). WB results established that the protein expression of FABP4 was also inhibited following adipocyte transfection with the *bta-miR-484* agomir (Figure 5D,E). On the contrary, the *bta-miR-484* antagomir promoted the expression of *CEBPA*, *LPL*, and *FABP4* genes, upregulated the expression of FABP4 protein, and increased lipid droplet accumulation (Figure 5F,J). These results suggested that *bta-miR-484* suppresses adipocyte differentiation.

### 2.5. RNA Sequencing Analysis

**Figure 6:**
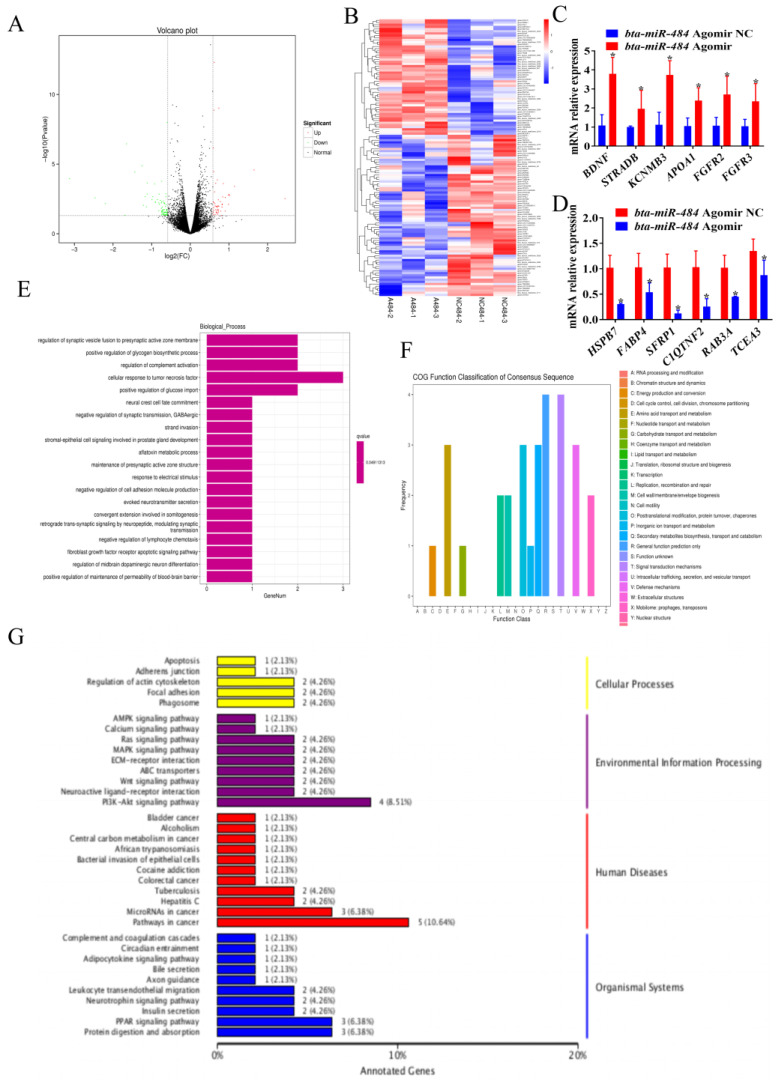
RNA sequencing analysis of *bta-miR-484* overexpression in adipocytes. (**A**) Differential expression volcano plot. (**B**) Heat map of differentially expressed mRNAs in control and *bta-miR-484* overexpressing adipocytes. A484 = “*bta-miR-484* agomir”, NC484 = “*bta-miR-484* agomir NC” (**C**,**D**) qPCR analysis of DEGs in control and *bta-miR-484*-overexpressing adipocytes. (**E**) GO enrichment histogram of differentially expressed genes. (**F**) COG annotation classification statistics of differentially expressed genes. (**G**) KEGG classification map of differentially expressed genes. Data are presented as mean ± SD. n = 3. * *p* < 0.05.

To further elucidate the role of *bta-miR-484* in adipocytes, we compared the mRNA expression profiles in control and *bta-miR-484* overexpressing adipocytes using RNA sequencing. In total, 51 upregulated and 77 downregulated genes were detected in adipocytes overexpressing *bta-miR-484* (Figure 6A,B). The RNA sequencing results were confirmed by using real-time PCR (Figure 6C,D). Gene ontology (GO) annotation indicated that differentially expressed genes (DEGs) were enriched in the positive regulation of glycogen biosynthetic processes, glucose import, and other biological processes (Figure 6E). The clusters of orthologous groups (COG) classification revealed that these DEGs were rich in amino acid transport and metabolism, posttranslational modification, protein turnover, chaperones, secondary metabolite biosynthesis, transport and catabolism, general function prediction only, signal transduction mechanisms, and other processes (Figure 6F). Kyoto encyclopedia of genes and genomes (KEGG) enrichment analysis indicated that these DEGs were enriched in the apoptosis (2.13%), AMPK (2.13%), calcium (2.13%), Ras (4.26%), MAPK (4.26%), Wnt (4.26%), PI3K-Akt (8.51%), adipocytokin (2.13%), insulin secretion (4.26%), and PPAR signaling pathways (6.38%) (Figure 6G).

### 2.6. Bta-miR-484 Targeted Binding of SFRP1

**Figure 7:**
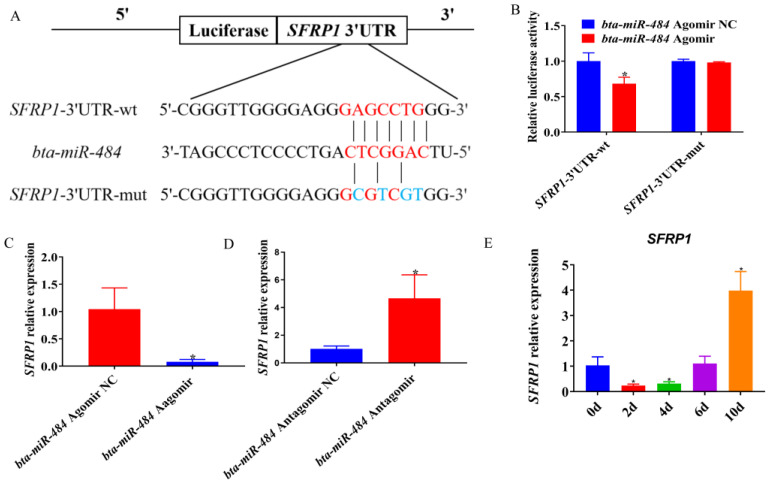
Verification of the *bta-miR-484* target gene, *SFRP1*. (**A**) *Bta-miRNA-484* and *SFRP1* 3’UTR binding sites. (**B**) Dual-luciferase reporter assay to evaluate the targeting relationship between *SFRP1* and *bta-miR-484*. (**C**,**D**) The expression level of *SFRP1* in adipocytes was detected after overexpression or inhibition of *bta-miRNA-484*. (**E**) Expression of *SFRP1* during induction of preadipocyte differentiation. Data are presented as mean ± SD. n = 3. * *p* < 0.05.

To further understand how *bta-miR-484* exerts its biological function, we utilized two bioinformatics tools, TargetScan and RNAhybrid, to predict its potential targets; we then combined these results with prior literature reports to screen for candidate target genes of *bta-miR-484*. We determined that the 3’UTR of *SFRP1* possessed a target sequence of *bta-miR-484*; further, the free-pairing energy between *bta-miR-484* and *SFRP1* was calculated as -38.6 kcal/mol. Moreover, RNA sequencing results demonstrated that overexpression of *bta-miR-484* down-regulated the expression level of *SFRP1*. Therefore, *SFRP1* was selected as a candidate target gene of *bta-miR-484*. To determine the targeted regulatory relationship between *bta-miR-484* and *SFRP1*, 3’UTR wild-type and mutant vectors of *SFRP1* were constructed, and the nucleotide mismatch between *bta-miR-484* and *SFRP1* was introduced by mutation (Figure 7A). A dual luciferase reporter assay demonstrated the cotransfection of *SFRP1* wild-type 3’UTR (*SFRP1*-3’UTR-wt) and *bta-miR-484* down-regulated relative luciferase activity. However, cotransfection of *SFRP1* mutant 3’UTR (*SFRP1*-3’UTR-mut) and *bta-miR-484* could no longer inhibit relative luciferase activity (Figure 7B). Furthermore, after the overexpression or inhibition of *bta-miR-484*, the expression levels of *SFRP1* and *bta-miR-484* exhibited opposite trends (Figure 7C,D). The expression of *SFRP1* was then detected at different time points during the induction of preadipocyte differentiation. With an increase in induction time, the expression of *SFRP1* initially decreased before increasing. (Figure 7E). Thus, *bta-miR-484* was determined to target *SFRP1* and negatively regulate its expression.

## 3. Discussion

The fundamental property of preadipocytes was their ability to proliferate and differentiate into mature adipocytes. *CEBPA* and *PPARγ* are key regulators of adipogenesis; in particular, the primary role of *CEBPA* in this process is to induce the expression of *PPARγ* [24]. Then, activated *PPARγ* acts as a transcription factor for *FABP4* and *LPL* genes expressed in mature adipocytes [25]. In the present study, isolated preadipocytes exhibited increased accumulation of lipid droplets and upregulated *CEBPA*, *FABP4,* and *LPL* gene expression following induction of differentiation. The temporal expression patterns of these genes were similar to those previously reported [26], indicating that the isolated preadipocytes were successfully transformed into mature adipocytes and suggesting that this model could be utilized in further bovine adipogenesis experiments. Subsequently, we evaluated the expression of *bta-miR-484* at different time points during bovine adipogenesis. Overall, the *bta-miR-484* expression levels initially increased before decreasing during adipocyte differentiation; this indicated that *bta-miR-484* may be involved in the regulation of adipogenesis.

Cell proliferation and apoptosis were essential biological processes. E-type cyclins bind to *CDK2* to promote G1/S transition during the normal cell cycle [27]. Additionally, *PCNA* plays a key role in DNA replication and replication-related processes [28]. Alternatively, activation of cysteine proteases (caspases) is the most widely recognized biochemical marker of early and late apoptosis. Specifically, the detection of *Caspase-3* and *Caspase-9* expression in cells is important for determining the induction of apoptosis [29,30]. BAX, a pro-apoptotic protein involved at the mitochondrial level, was a critical protein for enhancing apoptosis as it has the potential to induce necrotic cell death in some cases, even when caspase activation was inhibited [31]. In previous studies, *miR-484* has been shown to inhibit cell viability and promote apoptosis of granulosa cells by directly targeting Yes-associated protein 1 (*YAP1*) [32]. Additionally, *miR-484* has been observed to target CC chemokine ligand 18 (*CCL-18*) through the PI3K/AKT signaling pathway, thereby inducing G1 phase cell cycle arrest, inhibiting cell proliferation, and promoting apoptosis of gastric cancer cells [33]. These findings were consistent with the results of this study, which indicated that *bta-miR-484* overexpression in bovine adipocytes reduces cell viability, inhibited mRNA and protein expression of proliferation marker genes (PCNA and CDK2), induced G1 phase cell cycle arrest, promoted the mRNA expression of apoptotic marker genes (*BAX*, *Caspase 3,* and *Caspase 9)* mRNA, promoted the protein expression of BAX and Caspase 3, and increased the number of apoptotic cells. Based on these results, we conclude that *bta-miR-484* plays an important role in the proliferation and apoptosis of bovine preadipocytes.

Recently, various miRNAs have been implicated in the regulation of adipocyte differentiation. *miR-424(322)/503* targets γ-synuclein (*SNCG*), thereby regulating adipocyte differentiation [34]. Additionally, *bta-miR-149-5p* regulates bovine adipogenesis by downregulating the mRNA levels of adipogenic marker genes, such as *KLF6*, *ACSL1*, *SCD*, *SIK2,* and *ZEB1* in adipocytes [35]. Overexpression of *miR-19a* and *miR-19b* suppresses the expression of *PPARγ* and *CEBPA* in 3T3-L1 cells. [36]. Further, overexpression of miR-146b promotes the expression of genes associated with adipocyte differentiation, such as *CEPBA*, *PPARγ*, and *AP2* [37]. In the current study, we further explored the effects of *bta-miR-484* on adipocyte differentiation. Overexpression of *bta-miR-484* reduced lipid droplet formation and correspondingly decreased the expression of lipogenic markers (*CEBPA*, *FABP4*, and *LPL)*. Conversely, inhibition of *bta-miR-484* expression increased the accumulation of lipid droplet and promoted the expression of lipogenic markers. Simultaneous sequencing indicated that overexpression of *bta-miR-484* downregulated expression of *FABP4*, a gene positively associated with adipocyte differentiation, and upregulated apolipoprotein A 1 (*APOA1*), a gene negatively associated with adipocyte differentiation. Ultimately, these results suggest that *bta-miR-484* was involved in the differentiation of bovine adipocytes, and has a negative regulatory effect on the formation and lipid accumulation of bovine preadipocytes.

*SFRP1* was a Wnt antagonist involved in the regulation of adipogenesis and was expressed in both mouse and human mature adipocytes. Further, it has been determined to promote adipogenesis and inhibit the Wnt/β-catenin signaling pathway in vitro [38]. *SFRP1* deficiency can increase fat weight and adipocyte size, and was regulated during adipogenesis and obesity itself. *SFRP1* was upregulated in the early stages of obesity, thereby promoting adipose tissue expansion [39]. In addition, *SFRP1* affects the secretion of interleukin 6 (IL-6), monocyte chemoattractant protein-1 (*MCP-1*), and adiponectin, which were positively correlated with insulin sensitivity [40]. *SFRP1* has been previously identified as a direct target of *miR-542-3p* using a luciferase reporting assay. Additionally, *miR-542-3p* can alleviate the increase in adipogenesis after methotrexate treatment by inhibiting *SFRP1* [41]. However, the involvement of *SFRP1* as a direct target of *bta-miR-484* in the regulation of adipocyte differentiation remained unclear. Nonetheless, the current study identified *SFRP1* as a direct target of *bta-miR-484* using a double-luciferase reporter assay. Additionally, the mRNA expression of *SFRP1* was determined to be reduced in preadipocytes overexpressing *bta-miR-484*; in contrast, *SFRP1* expression was increased in adipocytes after *bta-miR-484* was silenced. Meanwhile, the corresponding RNA-seq results demonstrated that overexpression of *bta-miR-484* downregulated the expression of the *SFRP1* gene enriched in the Wnt signaling pathway. In addition, the expression of *SFRP1* and *bta-miR-484* showed an opposite trend with increasing time of adipocyte-induced differentiation. These results indicate that *SFRP1* is a target of *bta-miR-484*-mediated adipocyte differentiation. In conclusion, *bta-miR-484* negatively regulates adipocyte differentiation; this provides a novel molecular target for elucidating the regulatory mechanisms of adipocyte differentiation and adipogenesis in cattle.

## 4. Materials and Methods

### 4.1. Materials

Subcutaneous adipose tissue was collected from three premature calf. The adipose tissue was washed 4 times with PBS containing 2% penicillin-streptomycin (Pen-Strep, Hyclone, UT, USA). The visible nerves, blood vessels, and connective tissues on the surface were removed. Then, the adipose tissue was transferred to PBS containing 1% Pen-Strep and transported to the laboratory within 2 h at room temperature to isolate adipocytes. HEK293T cells were purchased from Fenghui Biological Corporation; these cells were resuscitated and cultured in the Key Laboratory of Ruminant Molecular Cell Breeding of Ningxia Hui Autonomous Region, before subculturing and cryopreservation.

### 4.2. Adipocyte Isolation, Culture and Differentiation

Preadipocytes were isolated from bovine adipose tissue using 1 mg/mL collagenase I (Sigma-Aldrich, St. Louis, MO, USA). Subsequently, these adipocytes were cultured in DMEM (Hyclone, Logan, UT, USA) containing 10% fetal bovine serum (FBS; Biological Industries, Kibbutz, Israel) and 1% Pen-Strep. After the adipocytes reached 100% confluence, the medium was replaced with induced differentiation medium. After 2 d of induction, the differentiation induction medium was discarded and maintenance medium was added. The maintenance medium was changed every 2 d before adipocytes were collected [42].

### 4.3. Oil Red O Staining

When adipocyte differentiation was complete, the culture medium was discarded, and the cells were washed with PBS three times and fixed with 4% paraformaldehyde at room temperature for 60 min, after which the excess paraformaldehyde solution was discarded and the cells were cleaned with 60% isopropanol. After ensuring the Petri dish was completely dry, the Oil Red O working solution was added and allowed to incubate at room temperature for 30 min. Next, Oil Red O was discarded, and the cells were washed four times with PBS. Then, images were captured using an inverted fluorescence microscope. Finally, Oil Red O was eluted from the stained cells with 100% isopropanol; then, absorbance was quantified at 510 nm using a multifunctional enzyme marker to analyze lipid droplet aggregation.

### 4.4. CCK-8 Assay and EdU Staining

Adipocytes were seeded in 96-well plates (Corning, New York, NY, USA) and allowed to adhere overnight. On the second day of incubation, adipocytes were transfected with *bta-miR-484* agomir, *bta-miR-484* agomir NC, *bta-miR-484* antagomir, or *bta-miR-484* antagomir NC; the medium was then replaced with fresh medium 6 h post-transfection. Subsequently, in each well, cells that were transfected for 6, 18, 30, 42, 54, 66, and 72 h were combined with 10 μL of cell counting kit-8 (CCK-8; Plomag, Beijing, China) reagents, and incubated for an additional hour. Sample absorbance was measured at 450 nm by using a multifunctional enzyme marker. A blank control was used to detect the CCK-8 absorbance of adipocytes without transfection. Finally, we analyzed the effect of *bta-miR-484* on adipocyte viability across these different time points of transfection.

### 4.5. Flow Cytometery

Adipocytes transfected with *bta-miR-484* agomir, *bta-miR-484* agomir NC, *bta-miR-484* antagomir, or *bta-miR-484* antagomir NC for 48 h were collected via trypsin digestion. Cell cycle phases were detected using the cell cycle and apoptosis analysis kit (Beyotime, Shanghai, China) and apoptosis was detected using the annexin V-FITC apoptosis detection kit (Beyotime). Flow cytometry was performed using an accuri C6 plus cytometer (BD Biosciences, San Jose, CA, USA), and the fraction of cells in each cell cycle phase and apoptosis stage were analyzed using FlowJo software (version v10.6.2; Tree Star Inc., Ashland, OR, USA).

### 4.6. RNA Extraction and Quantitative Real-Time PCR (qPCR)

Total RNA was harvested from adipocytes using Trizol reagent according to the manufacturer’s instructions (Invitrogen, Carlsbad, CA, USA). RNA concentration was then determined using multifunctional enzyme markers. Next, the total RNA was reverse-transcribed into cDNA using the PrimeScript RT kit (Takara, Kusatsu, Shiga, Japan); the cDNA obtained by replacing universal primers with specific stem ring primers was then used as a template for miRNA quantification. Then, qPCR was performed using a CFX96 system (Bio-Rad, Hercules, CA, USA) with SYBR Green Supermix (Takara). The sequences of the oligonucleotide primers used in this study were listed in Table S1. Glyceraldehyde-3-phosphate dehydrogenase (*GAPDH*)-normalized mRNA, *U6*-normalized miRNA, and 2^−ΔΔCT^ were used to analyze the relative mRNA abundance of each gene [43].

### 4.7. Western Blotting

Adipocytes were transfected with a *bta-miR-484* agomir, *bta-miR-484* agomir NC, *bta-miR-484* antagomir, or *bta-miR-484* antagomir NC before differentiation was induced. Adipocytes were collected using trypsin digestion (Hyclone) 2 d after transfection and 6 d after induction of differentiation. Proteins were isolated from adipocytes using a whole protein extraction kit (KeyGEN BioTec, Jiangsu, China) and total protein was quantified using a BCA protein quantification kit (KeyGEN BioTec). Denatured protein samples were then added to a 10% gel tank before electrophoresis at 80 v for 2 h, until they had transferred to the bottom layer of the separated gel. Using the wet transfer method, protein strips were electrotransferred to a PDVF membrane; then, the PDVF membrane was transferred to 5% skimmed milk powder and sealed for 2 h. Then, the milk powder was discarded, the membrane was washed 1–2 times with TBST, and the primary antibody was added before incubation overnight at 4 °C. Next, the membrane was washed 3 times with TBST for 10 min each time, the waste solution was discarded, the secondary antibody was added, and the membra ne was incubated for 2 h at a constant temperature. Finally, the PVDF membrane was treated with 200 µL of ultra-sensitive luminescent solution; the corresponding images were collected and stored by an imager. Image J software (version v10.6.2). was then used for quantitative analysis of protein bands. Antibody information is shown in Table S2.

### 4.8. RNA-Seq

Adipocytes transfected with *bta-miR-484* agomir or *bta-miR-484* agomir NC 48 h were collected using TRIzol (n = 3); then, second-generation transcriptome sequencing was performed at BioMarker Technology (Qingdao, Shandong, China) using an Illumina HiSeq TM sequencing platform. The primer used to verify the sequencing results were shown in Table S3. BMKCloud (www.biocloud.net, accessed on 21 June 2022) was used for differential expression screening, GO functional annotation, COG classification, and KEGG pathway enrichment analyses.

### 4.9. Target Gene Prediction

TargetScan databases (http://www.targetscan.org/vert_72/; accessed on 9 October 2021) were used to predict the *bta-miR-484* target mRNA, and the RNAhybrid database (https://bibiserv.cebitec.uni-bielefeld.de/mahybrid/; accessed on 9 October 2021) was applied to the analysis of *bta-miR-484* candidate targets and to calculate the free energy of interaction between *bta-miR-484* and these candidate genes [44]. At the same time, combined with the literature reports, the candidate target genes of *bta-miR-484* were screened.

### 4.10. Dual Luciferase Reporter Gene Assay

Dual luciferase reporter vector with *SFRP1*-3’UTR-wt and *SFRP1*-3’UTR-mut binding sites were constructed by General Biol (Anhui, China). *SFRP1*-3’UTR-wt or *SFRP1*-3’UTR-mut were co-transfected with *bta-miR-484* agomir or *bta-miR-484* agomir NC into HEK293T cells cultured to 70% confluence. Lipofectamine 3000 (Invitrogen) was used as the transfection reagent in this protocol. Then, these HEK293T Cells were harvested 48 h after transfection, and the relative fluorescence activity was measured using the Dual-Glo Luciferase Assay System (Promega, Madison, WI, USA) according to the manufacturer’s instruction.

### 4.11. Statistical Analyses

Differences between the groups were analyzed using a Student’s *t*-test when comparing two groups or a one-way analysis of variance (ANOVA) when comparing two groups. All data were presented as mean  ±  standard deviation (SD). * *p* < 0.05 was considered statistically significant. GraphPad Prism 7 (GraphPad, San Diego, CA, USA) was used for graphical representation of the data.

## 5. Conclusions

In the present study, we performed a series of assays to characterize and evaluate the function of *bta-miR-484*; these suggested that *bta-miR-484* targets *SFRP1* and regulates proliferation, differentiation, and apoptosis. Overall, these results provide a strong reference for further elucidation of the mechanism of action of *bta-miR-484* in bovine adipogenesis.

## Data Availability

The RNA-seq data (GSE238109) were uploaded to GEO (https://www.ncbi.nlm.nih.gov/gds/?term=GSE238109, accessed on 31 July 2023).

## Supplementary Materials

The following supporting information can be downloaded at: https://www.mdpi.com/article/10.3390/ijms241612710/s1.
