# Supplementary material for: Bta-miR-484 Targets SFRP1 and Affects Preadipocytes Proliferation, Differentiation, and Apoptosis
Source: Int J Mol Sci. 2023 Aug 11;24(16):12710. doi: 10.3390/ijms241612710 (PMC10454478; doi:10.3390/ijms241612710)
Supplement: Supplementary file 1 [file ijms-24-12710-s001.zip › Table S1.pdf]

Table S1. Oligonucleotide primer sequences used for quantitative real-time polymerase chain reaction.

Gene	Primer sequence	Annealing temperature/℃
bta-miR-484	RT：GTCGTATCCAGTGCGTGTCGT<br>GGAGTCGGCAATTGCACTGGATACGACATCGGGAG	-
bta-miR-484	F：GAATACTCAGGCTCAGTCCC<br>R：CAGTGCGTGTCGTGGAGT	60
U6	F：GCTTCGGCAGCACATATACTAAAAT<br>R：CGCTTCACGAATTTGCGTGTCAT	60
SFRP1	F:GCTCAAGTGCGACAAGTTTC<br>R:GCTCAATGATGGCTTCAGAC	60
C/EBPα	F：TGGACAAGAACAGCAACGAG<br>R：TTGTCACTGGTCAGCTCCAG	60
FABP4	F：AAGTCAAGAGCATCGTAA<br>R：CCAGCACCATCTTATCAT	60
LPL	F: ACGATTATTGCTCAGCATGG<br>R: ACTTTGTACAGGCACAACCG	60
GAPDH	F：ACTCTGGCAAAGTGGATGTTGTC<br>R：GCATCACCCCACTTGATGTTG	60
PCNA	F：TCCAGAACAAGAGTATAGC<br>R：TACAACAGCATCTCCAAT	60
CDK2	F：TTTGCTGAGATGGTGACCCG<br>R：TAACTCCTGGCCAAACCACC	60
Bax	F：GAGATGAATTGGACAGTAAC<br>R：TTGAAGTTGCCGTCAGAA	60
Caspase 3	F：CAGCGTCGTAGCTGAACGTA<br>R：CCAGAGTCCATTGATTTGCTTCC	60
Caspase 9	F：TGGTGGTCATCCTGTCTC<br>R：CATCCATCTGTGCCATAAAC	60
