# Supplementary material for: Bta-miR-484 Targets SFRP1 and Affects Preadipocytes Proliferation, Differentiation, and Apoptosis
Source: Int J Mol Sci. 2023 Aug 11;24(16):12710. doi: 10.3390/ijms241612710 (PMC10454478; doi:10.3390/ijms241612710)
Supplement: Supplementary file 1 [file ijms-24-12710-s001.zip › Table S2.pdf]

Table S2. antibodies information of Western blot.

Name of antibody	Dilution rate	Company	Product code
PCNA	1:500	Sangon Biotech	D220014-0025
CDK2	1:500	Abways	cy5020
FABP4	1:500	Abways	CY6768
Caspase-3	1:1000	Beyotime	AC030
Bax	1:1000	Abways	CY5059
GAPDH	1:2000	Abways	AB0036
Goat anti-Rabbit IgG	1:20,000	Sangon Biotech	D111018
