# Supplementary material for: Bta-miR-484 Targets SFRP1 and Affects Preadipocytes Proliferation, Differentiation, and Apoptosis
Source: Int J Mol Sci. 2023 Aug 11;24(16):12710. doi: 10.3390/ijms241612710 (PMC10454478; doi:10.3390/ijms241612710)
Supplement: Supplementary file 1 [file ijms-24-12710-s001.zip › Table S3.pdf]

Table S3. Primers information of qPCR to verify sequencing results.

Gene	Primer sequence	Annealing temperature/℃
HSPB7	F：CCCTGGGAGACGCCTATGA<br>R：TGTGGTTGTTGGAGGTGGTG	60
SFRP1	F：GCTCAAGTGCGACAAGTTTC<br>R：GCTCAATGATGGCTTCAGAC	60
C1QTNF2	F：CTCAGGAATGGTGGGAAGAA<br>R：CACCGCTGACACCTTTGG	60
RAB3A	F：TTTCCTCTTCCGCTATGC<br>R：GGACTCCTCGTTGGTAATG	60
TCEA3	F：AGATCAAACAGCAGCAAATCA<br>R：TGTAATCATCATCCGCCTTC	60
FGFR2	F：AACTGTATATGATGATGAGAG<br>R：ATCCAAGTCTTCTACCAA	60
BDNF	F：CATGGGACTCTGGAGAGCAT<br>R：CAAAGGCACTTCACTGCTGA	60
STRADB	F：GCAGGTTCCTTTCCAAGAC<br>R：TATTTACGGTGCGAGTTCC	60
KCNMB3	F：TTCTCGGTCCTAATGTTCT<br>R：ACAGTCCGTCCAGTCATC	60
APOA1	F：ACCGTGTATGTGGAAGCAATCAAG<br>R：TCCCAGTTGTCCAGGAGTTTCAG	60
FGFR3	F：GTCCTTGGAGTCCAGCTCATC<br>R：TATCCGTGGCGTCATCTTTC	60
